# Fast prototype selection algorithm based on adjacent neighbourhood and boundary approximation

**DOI:** 10.1038/s41598-022-23036-9

**Published:** 2022-11-22

**Authors:** Juan Li, Cai Dai

**Affiliations:** 1 College of Distance Education, Shaanxi Normal University, Xi’an, 710062 Shaanxi China; 2 College of Computer Science, Shaanxi Normal University, Xi’an, 710110 Shaanxi China

**Keywords:** Computer science, Information technology

## Abstract

The unceasing increase of data quantity severely limits the wide application of mature classification algorithms due to the unacceptable execution time and the insufficient memory. How to fast incrementally obtain high decision reference set and adapt to incremental data environment is urgently needed in incremental environments, large dataset, etc. This paper proposes a novel prototype selection algorithm by integrating the strategies between condensing method and editing method. To an unlearned pattern, this algorithm extends the references scope from its single nearest neighbour to its *k* nearest neighbourhood that can expand the judgment information to obtain its detailed neighbour relationship. Then a pattern was determined whether it is a prototype using its neighbour relationship and classification boundary asymptotically strategy. To maintain the higher reference set, this algorithm periodically updates those prototypes that locates in the non-boundary zone or is long-time unlearned. The empirical study shows that this algorithm obtains the smaller and higher boundary prototypes without decreasing classification accuracy and reduction rate than the compared algorithms.

## Introduction

Existing efficient classification algorithms has introduced unacceptable space-time consumption when deal with large dataset, not to mention data stream^1^. How to remove redundant patterns, retain the high reference patterns of efficient classification contributions, and then achieve the goal of reducing the data scale and the classification executing time has become a research hotspot in the field of pattern classification. Therefore, an effective processing strategy was proposed, that is prototype selection^2^, is the necessary reduction of the original data set based on some techniques. The reference prototype set that can reflect the distribution and classification characteristics of the original data can be obtained by using prototype selection. The fast running time can be obtained owing to the strategy of prototype selection can reduce the sensitivity of data scale and noise without losing classification accuracy. Then the above problem of unacceptable space-time consumption has been solved slightly better. Meantime, prototype selection algorithms themselves have many inner drawbacks^3^ such as pattern reading sequence, outlier and noise, etc, which is noteworthy exploring deeply. Therefore, in order to overcome the sequence sensitivity of Condensed Nearest Neighbor (CNN)^4^ and obtain the reference prototypes that is nearby the classification boundary more efficiently, based on CNN algorithm, this paper proposes some ideas such as nearest neighbour neighbourhood and classification interval distance, and classification boundary approximation.

Then a fast prototype selection algorithm based on classification neighbourhood boundary approximation (PSNB) is designed taking full advantage of the simple incremental selection strategy of CNN and these proposed ideas. The two main processes of PSNB are selection process and update process respectively.

The selection process is an actual prototype learning process which is the process of deciding which patterns are retained as prototypes. Different with the single prototype-judging mechanism of CNN, PSNB enlarges the reference range from its nearest neighbour to the whole relationship of its several nearest neighbours to each coming pattern. Through analysis these extend related neighbours, the whole neighbour relationship can well reflect the location, the peripheral situation, outlier, etc. In order to utilize these concepts effectively, PSNB extends the scale of the reference prototypes, formulates the changes of the neighbourhood class label and their classification boundary distance as a prototype selection criterion to evaluate whether an unlearned pattern is selected as a prototype. The second process, group updating process similar with Edited Nearest Neighbor (ENN)^5^, is basically to continuously maintain the high reference value of the prototype set through eliminating some prototypes which are inappropriate for the classification boundary optimization and the low interference influence. Similar with the updating mechanism of some incremental algorithms^6^, PSNB achieves the group update effect by utilizing the updating threshold to guide whether a prototype should be removed from the prototype set. The updating threshold value is self-adaptive generated taking into account of the variation of the classification neighbourhood distance and the class distribution of the prototype.

The major contributions of this paper are as follows: (1) PSNB can overcome the shortcomings of CNN without increasing the processing complexity. (2) References the group updating learning strategy of ENN, PSNB not only achieves the prototype selection, but also obtains the location and class label information at the same time, and then ensures the efficient selection of high-classification contribution boundary decision prototypes. (3) Taking account of the location and inactive state, PSNB designs the update strategy in prototype dynamic update process, and overcomes the unicity of traditional incremental update. (4) PSNB can tackle the problems with imbalanced-class and similar interclass-patterns. The weakness of PSNB is the larger computation cost than CNN because it needs to judge complex neighborhood information for prototype selection and maintains the high classification ability of the prototype set.

The organization of the remaining paper is reproduced below. Section “Related work” introduces existing research, including those prototype selection algorithms, some characteristic technologies of classification boundary and decision pattern. Section “A fast prototype selection algorithm based on adjacent neighbourhood and classification boundary approximation” describes our new integration strategy to prototype selection algorithms. Experimental evidence on the effectiveness of the proposed algorithm is provided in Section 4 through a series of experiments and analyses. Finally in Section 5, the conclusions are briefly summarized.

## Related work

The related techniques of prototype selection algorithms, nearest homogeneous and heterogeneous neighbour strategies are stated in this section.

### Prototype selection algorithm

The goal of prototype selection is to reduce the scale of reference set while maintaining classification accuracy. As an independent algorithm, prototype selection can obtain the higher and smaller reference set. As soon as the prototype selection algorithm was put forward, it gained great development and produced a lot of research results. There are three types of prototype selection algorithms: condensation methods, edition methods, and hybrid methods. Among them, CNN and ENN were first proposed based on prototype selection. As the first condense method, the disadvantage of CNN is sensitive to the pattern sequence and the noise influence, and there are many redundant prototypes in the final decision set.

In order to overcome these problems of CNN, a series of improved algorithms were developed based on CNN. RNN (Reduced Nearest neighbour Rule)^7^ focuses the updating process to overcome the disadvantages of only selecting without deleting to prototype, etc. Fast Condensed Nearest neighbour algorithm (FCNN)^8^ focuses on reducing the pattern sequence sensitivity and retrieving these classification boundary prototypes as far as possible. Mutual neighbourhood Value algorithm (MNV)^9^ applies mutual neighbour value to reduce the sequence sensitivity of the benchmark algorithm. Except for the majority class information of the nearest neighbors of a pattern, the second majority class information, the quantity and distribution information on the k-neighborhood in the discrimination classes was efficiently utilized in DC-LAKNN^10^. Furthermore, based on CNN, POC-NN^11^ algorithms, Multiedit Condensing (MCNN)^12^ and New Rank Methods^13^, which introduced sequencing, boundary approximation and other ideas, a self-guiding criterion is proposed for the actual selection, and can eliminates the need for tuning an external user parameter that the classical methods hold can remove noise to a certain extent, reduce the impact of reading sequence, and improve the efficiency of high-classification value archetypes, but with increased computational complexity and high space-time cost. In addition, the CNN family algorithms still fail to reduce noise sensitivity.

ENN and its improved family algorithms^14,15^ had been designed to remove noise and clean up the patterns of different categories corner that achieve the goal of representative points choice, mainly to eliminate the noise from the original sample concentration, etc. As static well-known prototype selection algorithms, they cannot work well on large-scale data environment.

In addition, some explorations focus on optimizing traditional algorithms around improvement way and hybrid strategy to solve their imperfect application problems in prototype reduction. kNNc^16^ adopts a three-stage cycle guidance mechanism to tackle KNN’s inherent drawbacks based on combining the classification accuracy of retaining all the training set and the time efficiency of dynamic prototype set. The family of new rank methods^17^ based on *k* Farthest Nearest Neighbour or *k* Nearest Enemy are proposed to cope with the classical rank influence of KNN and avoid outliers. Differing from conventional prototype selection, an improved K-Nearest Neighbor algorithm (IKNN_PSLFW)^18^ combines prototype selection and local feature weighting. The prototype selection of IKNN_PSLFW divides the orginial training set into pure subsets, preserve the information of subsets, and selects the representative instances as prototypes from each pure subsets. Global density-based instance selection (GDIS)^19^ adopted global density and relevance function to determine the importance of patterns and select the relevant patterns. NearCount^20^ used the corresponds cited counts of an instance to evaluate its importance and selected critical instances based on the cited counts of nearest neighbors. Cluster-oriented instance selection (CIS)^21^ runned unsupervised K-Means Clustering (KMC) algorithm to identify clusters in the training instances and using distance based measures to select instances from the centers and the borders of the clusters. The evolutionary prototype methods,such as ANN-KNN^22^, and DWP^23^, etc. are introduced to improve the performance of the condensing KNN algorithm or NN classifier, handle the class imbalance problem, etc. Some ensemble algorithms^24,25^ using boosting methods are proposed to improve the performance of a single classifier.

Partition strategy and boundary location identification are merged into the condense process in order to obtain the efficient decision prototype set from the original dataset. Prototype’s Front Propagation method^26^ is adopted to determine the boundary between classes. Fast partitioning processes were proposed in RSP3^27^. Two PSP3 variations choose a pair of distant-enough instances to instead of generally computing the actual diameter of a non-homogeneous subset that maintains an approximations convex hull distribution. The clustering strategy and boundary selection process are merged in to reduction algorithm, such as PSC^28^, Cluster evolutions^29,30^, etc.

Therefore, how to reduce the sensitivity of the traditional incremental prototype selection algorithm to the pattern reading sequence and abnormal nodes has become the research hotspot of incremental prototype selection algorithm, and also the main problem of this paper. To this end, it is necessary to integrate the processing strategies of CNN and ENN. The high-quality prototype set can be obtained through some improved group learning method, and then the influence of noise and abnormal can be reduced on the basis of maintaining incremental learning.

### Nearest homogeneous neighbour and nearest heterogeneous neighbour

In general, patterns nearby classification boundary zone are considered to have high classification contribution, while patterns at the distribution center and even far from the classification boundary are considered to have low classification contribution. Therefore, finding prototype nearby the classification boundary prototype is always a key to obtain the high classification decision prototypes.

Without considering noise, the line or plane connecting the patterns of different class labels must intersect with the classification boundary. Patterns of different class labels closest to the cross line or cross plane are located at the classification boundary and have higher classification contribution value. At the same time, the selection of all distance cross boundary samples reflects the idea of heterogeneous neighbours, which can maintain the classification boundary characteristics of the original dataset. In static environment, boundary patterns of all heterogeneous pattern pairs can be obtained by traversing the whole dataset several times, then the finial prototype set can be obtained by selecting the boundary patterns with the smallest cross attachment distance. For example, Generalized Nearest neighbour algorithm (GNN)^9^ introduces the homogeneous-heterogeneous neighbours and utilize these related nearest prototypes to overcome the shortcoming of CNN in using only the homogeneous neighbour. In PSC, the whole dataset is divided into homogeneous or heterogeneous pattern clusters by clustering method. For homogeneous pattern clusters, the central sample of the cluster is generated as the prototype. For heterogeneous pattern clusters, the patterns are selected as prototypes based on the principle of nearest heterogeneity. The selection principle reflects the cross-selection mechanism guiding the alternating acquisition of nearest heterogeneous and nearest homogeneous neighbours. TRKNN^31^ and its impoved hybrid algorithm^32^ shows the template reduction idea based on KNN. The basic idea is to define a nearest neighbour ‘chain’ that is characterized by the alternating appearance of the nearest heterogeneous neighbours and the distance between adjacent patterns keeps shrinking. By setting a cutoff value for the distances among the chain, the patterns near the classification boundary can be effectively separated based on the chain condense process and the interior patterns are removed.

Based on the margin theory that the patterns close to the classification boundary play far more essential role than those within the core distribution zone of the class, Fuzzy boundary region^33^ is adopted to express the boundary region by the linguistic term, possibilistic C-Means clustering^34^ and fuzzy set operators such as t-norm, s-norm and lp,1-norm grouping^35^. The conditional fuzzy C-Means clustering was used to locate the prototypes for nearest classifier within the boundary region with the aid of information about the defined boundary region.

Although the above algorithms can be applied to static environment, they cannot be implemented efficiently in incremental or big data environment, especially in incremental learning to adapt to the dynamic changes of data distribution. In order to achieve the goal to obtain better classification decision prototypes in the incremental learning, the new prototype set is unable to select from the whole dataset one time, instead, should be selected and optimized out step by step.

## A fast prototype selection algorithm based on adjacent neighbourhood and classification boundary approximation

In order to simply the description of the proposed algorithm, this paper adopts the following notations. Let $D=\lbrace { x_i=(x_{i1},x_{i2},...,x_{id})\vert i=1,2,...\rbrace }$ be a set of patterns as *d*-dimensional vectors in $R^n$ and $C=\lbrace {c_i,c_2,...,c_m\rbrace }$ as the class label information set, where *m* is the number of different class labels. Given a labeled training set $TR={ \lbrace (x_i,c_i)\vert x_i\in D, c_i\in C,i=1,2,...,n \rbrace }$ that contains some irrelevant even interference information, such as outlier, redundancy, noise, etc. Let $TP \subset TR$ the reduced set (Training Prototype Set) be the final reference set obtained by *TR*. Let *n* be the number of patterns of *TR* and *N* be the number of prototypes of *TP*. The testing set *TS* that includes some patterns to verify the validity of some correlation algorithms has the homogeneous structure with *TR*. Let $\vert S\vert$ as the scale of a set *S*. Let *label*(*x*) as the class label of a pattern or a prototype. Let *d*(*x*, *y*) as the Euclidean distance between *x* and *y* whether *x* and *y* are the pattern or prototype. Let *d*(*x*, *S*) as the Euclidean distances between *x* and a set *S*. Let *PS* be represented as the extension information of *TP*.

In the case of $TP\not =\emptyset$, $\forall x\in TR$ and $\forall p\in TP$, if $\min \limits _{i}d(x,TP)$, then *p* is the *i*-th nearest neighbour of *x* (where,$i=1,2,...,\vert TP\vert$), denoted as $p=TP(x,i)$. The prototype selection criterion of CNN only utilizes the class label information of its nearest neighbour prototype and each learning pattern. When their class labels are different, the learning pattern is selected as a prototype. Otherwise the pattern is discarded. Utilizing only the single class label information must be discarded some important neighbour information. To deal with the disadvantage of CNN, it’s necessary to extend the neighbour relationship between unlearned pattern *x* and its neighbours. So, in this paper, the new selection criterion is introduced that extends the information of its nearest neighbourhood besides the prototype itself.

### Definition 1:

Nearest neighbourhood (*NN*). To $\forall x\in TR$ , $\exists$ $k$ nearest neighbour prototypes of *x* in *TP*. The zone formed by the *k* prototypes as boundary points are named as the *k*-nearest neighbourhood of *x* or the nearest neighbourhood of *x* for short $NN(x)=\Omega (\bigcup \limits _{i=1}^{k}TP(x,i))$. Let *NN*(*x*, *i*) be the *i*-th ($i=1,2,...,k$) pattern or prototype that makes up the nearest neighbourhood of *x*. For the convenience of calculation, $k=3$ is selected in this paper.

However how to deal with the situation for the same class label among the learning pattern and its neighbours? How to use the class label information to recognize the detail location of a pattern or its *k* nearest prototypes? So further strengthening the near neighbour relationship is necessary. Then the pattern *x* is inserted into its neighbourhood as its self-nearest neighbour to make up its extended nearest neighbourhood that is introduced in Definition 2.

### Definition 2:

Extended Nearest neighbourhood (*EN*). A pattern *x* is seen as its self-nearest neighbour and inserted into its neighbour group to form the extend neighbours of *x*. The region formed by the extended neighbours is the extended nearest neighbourhood of *x*, denoted as $NN(x)=\Omega (x,\bigcup \limits _{i=1}^{k} TP(x,i))$. Let *EN*(*x*, *i*) be the *i*-th ($i=1,2,...,k$ ) pattern or prototype that makes up the extend neighbourhood of *x*. The value of *k* is 4 at here.

### Definition 3:

Classification neighbourhood (*CN*). To $\forall x\in TR$, the classification boundary must pass through *NN*(*x*) or *EN*(*x*) if and only if *x* has different types of *k* nearest neighbours in *TP* or extended nearest neighbours, claimed the neighbourhood of *x* is a classify neighbourhood, denoted as *CN*(*x*). Let *CN*(*x*, *i*) be the *i*-th ($i=1,2,...,k$) pattern or prototype that makes up the classification neighbourhood. When *NN*(*x*) is classification neighbourhood, the value of *k* is 3. When *EN*(*x*) is classification neighbourhood and *NN*(*x*) is not a classification neighbourhood, the value of *k* is 4.

### Definition 4:

NON-Classification neighbourhood (*NCN*). To $\forall$x$\in TR$, *NN*(*x*) is a non-classification neighbourhood *NN*(*x*) if and only if *EN*(*x*) is not a classification neighbourhood and each *EN*(*NN*(*x*, *i*)) of its neighbours is still not classification neighbourhood simultaneously. To $\forall NCN(x)$, each *NN*(*x*, *i*) ($i=1,...,k$, $k=3$) is not a classification boundary prototype, and has little contribution for classification.

In general, the judgment range of the nearest neighbour neighbourhood of *x* can be further extended, and all the descendants of the nearest neighbour neighbourhood of *x* can be traversed forward. Then through analysis and judgment the neighbour relationship, deciding whether *x* is a prototype. For example, if all of neighbourhoods of its offspring have the different class label with *x* and *x* is not a new class pattern, *x* is noise. If none of them is classification neighbourhood, it means that the neighbours of *x* are located in the distribution center and the overall classification contribution is little. If *NCN*(*x*), the prototypes of *NN*(*x*) has little contribution of classification. Likewise, if *x* can approach the classification boundary, it can be selected as a prototype. In this paper, the main goal of the proposed algorithm is to simplify the patterns of the center region. Similarly, to reduce the execution cost, it only traverses forward to the nearest neighbours of the nearest neighbours of *x*.

### Definition 5:

Classification neighbourhood Distance. $\forall CN(x)$, $\forall x\in TR$ or $\forall x\in TP$, the average distance between the neighbours of different class labels of *CN*(*x*) is defined as the classification neighbourhood distance of *CN*(*x*), denoted as *dh*(*CN*(*x*)).

The distance of each classification neighbourhood *CN*(*x*) can be computed by

1 $$\begin{aligned} dh(CN(x))=\frac{\sum \limits _{i=1,j=1}^{i=k,j=k,i\not =j} f(CN(x,i),CN(x,j))*d(CN(x,i),CN(x,j))}{\sum \limits _{i=1,j=1}^{i=k,j=k,i\not =j} f(CN(x,i),CN(x,j))}\end{aligned}$$

where

2 $$\begin{aligned} f(x,y)=\left\{ \begin{array}{rcl} 1 &{} label(x)\not =label(y)\\ 0 &{} label(x)=label(y)\\ \end{array} \right. \end{aligned}$$

Intuitively, if a pattern or prototype *x* has a classification neighbourhood *CN*(*x*), that indicates the nearest neighbours of *x* are nearby the classification boundary. In the incremental environment, the prototypes will approach the classification boundary step by step along with the shrinking classification neighbourhood of *x*. Then the approximating classification neighbourhood formed by *x*’s nearest neighbours can serve nicely to show the original classification boundary of the partial pattern set. The smaller the classification neighbourhood volume is, the smaller the classification boundary distance among patterns is, and vice versa. To a certain extent, the size of classification neighbourhood can also be reflected in the distance between samples that constitute the classification neighbourhood. Considering the difficulty calculation of multi-dimensional space area, this paper simplifies the calculation of classified neighbourhood area to calculate the average distance between neighbours of the classification neighbourhood.

### Definition 6:

Classification Decision Pattern. To $\forall x\in TR$ or $\forall p\in TP$, if $\exists CN(x)$ or $\exists CN(p)$ and there is $\min (dh(CN(x)))$ or $\min (dh(CN(p)))$, *CN*(*x*) or *CN*(*p*) is defines as the minimum classification neighbourhood. *x* or *p* is considered to be located at the classification boundary and called as the classification decision pattern.

And again, we’re going to study the situation that *NN*(*x*) is not a classification neighbourhood and *EN*(*x*) is. For further treatment the situation, *EN*(*x*) can be divided into three sub-neighbour neighbourhoods containing *x*, which is $NN_i(x)=\Omega (x,\bigcup \limits _{j=1,j\not =i}^{k}TP(x,j))$ $(k=3)$ respectively. By judging each subset case one by one, the result of whether *x* is a prototype or not can be obtained. For the convenience of expression, it is used to express as $CN_i(x)$ when $NN_i(x)$ is classification neighbourhood.

### Definition 7:

Classification Boundary Approximate. To $\forall x\in TR$, the local classification boundary that is represented by $CN_i(x)$ is superior than the original one that is represented by *CN*(*x*) if $\exists CN_i(x),i=1,...,k$ and $dh(CN_i(x))<dh(CN(x))$. In other words, the local classification boundary that is represented by $CN_i(x)$ is more approximate the original classification boundary.

The process of prototype selection is the process of continuously selecting the classification decision patterns with high classification contribution as prototypes and retaining the prototypes with high classification contribution. For non-incremental learning algorithm, it is easy to find the minimum classification neighbourhood of *x* or *p* by judging the nearest-neighbours relationship of any pattern *x* or prototype *p* and obtain the classification decision pattern or prototype. However, an incremental learning algorithm cannot obtain the actual minimum classification neighbourhood by obtaining the nearest neighbour relations of all patterns one time due to those continuous incoming data, but only uses the continuous approximation approach to gradually select the relatively optimal classification decision patterns or prototypes, and adopt the constant update way to eliminates those prototypes with low classification contribution.

An unlearned pattern *x* can be selected as a prototype in three situations:

- *x* belongs to a new class;
- *label*(*x*) is the same as one of its some nearest neighbours. If and only if $\exists dh(CN_i(x))<dh(CN(x))$ ($i=1,2,3$), *x* is selected as a prototype;
- *label*(*x*) is different from the class labels of its all neighbours, but the same with one of its neighbours’ neighbours. If $\exists dh(CN(\Omega (x,NN(TP(x,i))))\le dh(CN(TP(x,i)))$ $(i=1,2,3)$, it indicates that $CN(\Omega (x,NN(TP(x,i)))$ is closer to the boundary of the category than *CN*(*TP*(*x*, *i*)), then *x* is selected as a prototype.

**Figure 1:**
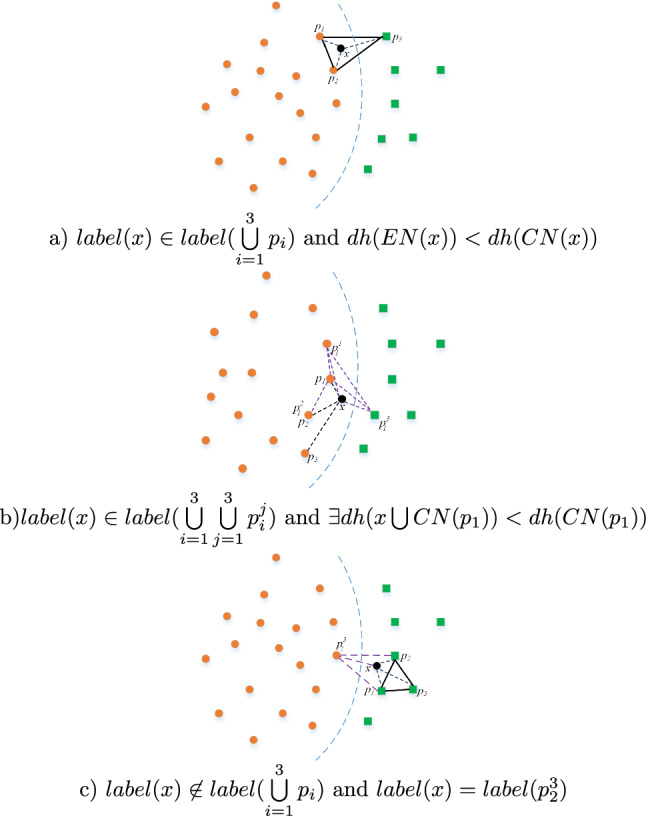
Prototype selecting schematic diagram.

Situation 2 and Situation 3 can help find outliers and update some related outliers to reduce their influence. Addition, Situation 3 can also be beneficial to reduce the influence of noise. Remaining process schematic of prototype selection is shown in Fig. 1 except for Situation 1.

For Fig. 1, *x* is an unlearned pattern, $p_i (i=1,...,k)$ is its *i*-th nearest prototype, $p_i^j(j=1,...,k)$ is its *i*-th nearest prototype’s *j*-th nearest prototype, and $\bigcup \limits _{i=1}^k\bigcup \limits _{j=1}^kp_i^j$($k=3$) are all its offspring nearest prototyps. The second situation of prototype selection is vividly represented in Fig. 1a–b, which Fig. 1b is a special case of Fig. 1a. For Fig. 1a, *NN*(*x*) and *EN*(*x*) are both classification neighbourhoods. *X* is clearly nearer by the boundary than $p_1$ and $p_2$. So *x* is selected as a prototype. For Fig. 1b, *EN*(*x*) is homogeneous classification neighbourhood, but $label(x)\not =label(p_1^3)$. $dh(x\bigcup CN(p_1))<dh(CN(p_1))$ can be obtained, then *x* is selected as a prototype. For Fig. 1c, although *x* has the different class label with $\bigcup \limits _{i=1}^3(p_i)$, it has the the same class label as one of its offspring nearest prototypes$\bigcup \limits _{i=1}^{3}\bigcup \limits _{j=1}^{3}p_i^j$. $dh(x\bigcup p_1\bigcup p_2\bigcup p_2^3)<dh(p_1\bigcup p_2\bigcup p_2^3)$ can be obtained and *x* is deemed to promotes the boundary approximation. So *x* is selected as a prototype.

**Figure 2:**
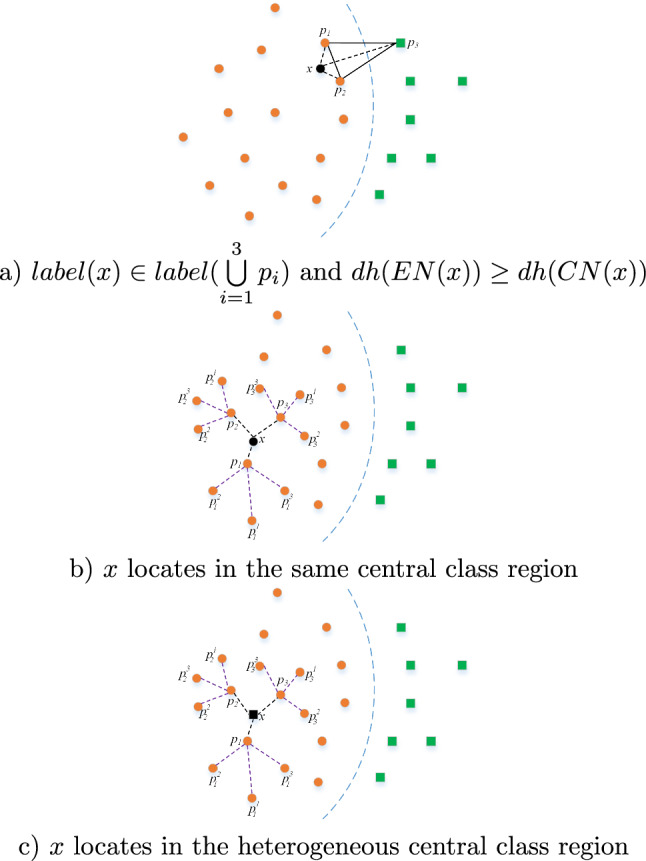
Prototype discarding and updating schematic diagram.

Excepting the above three situations, there are other situations during prototype selection process that *x* can be discarded. The profile process is shown in Fig. 2. Figure 2a illustrates the discard processing about *x* is far away the boundary. The classification neighbourhood distances *dh*(*EN*(*x*)) is larger than the original distance *dh*(*CN*(*x*)) when *x* has the same class label with one of its nearest prototypes. It is mean that *x* is inferior to $p_1$ and $p_2$, and can be discarded. Figure 2b illustrates the sparse processing about the central class region. *NN*(*x*) and $\bigcup \limits _{i=1}^k\bigcup \limits _{j=1}^k{p_i^j}$ are homogeneous class label that is the same as *label*(*x*). *x* is deemed to locate at the central class region and *NCN*(*x*) too. So *x* is discarded and the updating status of $\bigcup \limits _{i=1}^3{p_i}$ is marked. For Fig. 2c, *x* can be deemed as noise if it is different class label with its offspring prototypes.

In the traditional prototype selection algorithm, only a few relevant prototype information is utilized, but the relevant prototype information, such as location and class label, etc., is ignored. In this paper, a quaternion structure $PS=<(p,index,age,status)>$ is used to represent the neighbour relationship between each prototype and its boundary nearest neighbours for *TP*, in which *ps*(*p*) represents a prototype itself, *ps*(*index*) represents the index of $\bigcup \limits _{i=1}^{k}TP(x,i)$($k=3$) in *TP*, *ps*(*age*) represents the age time of the prototype was taken as one of *k*-nearest neighbours, and *ps*(*status*) represents the value of its updating status.

### Main strategy

This paper comprehensives the selection principles among CNN family algorithms and some reduction algorithms based on classification boundary and partition, effectively use the cross characteristic between the near neighbourhood and classification boundary, puts forward the incremental learning strategy and set the update mechanism. Based on the designed learning and update process, the proposed algorithm that can effectively avoid the inherent shortages of expensive running cost and noise sensitive about CNN is developed. Meanwhile, the proposed algorithm can be effectively applied to large-scale data reduction needs.

PSNB adopts the idea of expanding learning, no longer follow the thought of CNN that just utilizes the characteristics of a pattern itself for prototype dynamic learning. It is clear that those near the boundary patterns are important than the others and the difference between noise and the nearby patterns is large. Based upon the clear reorganization, PSNB expands the nearest prototype of each unlearned pattern to the nearest neighbourhood that is formed by the neighbour prototypes and judges the neighbour relationship characteristics of each unlearned pattern and its neighbourhood, then performs collective dynamic learning.

PSNB does not add complex prototype selection judgment process as comparing with CNN. Just in the process of prototype selection, it changes the judging basis of prototype selection based on the class information between each unlearned pattern and its nearest prototype. The nearest prototype of each pattern is extended its *k* neighbour prototypes in this paper. Based on the extended neighbour prototypes, the neighbourhood is built, and the neighbour relationship of the neighbourhood is obtained. The algorithm simply determines the neighbour relationship, such as class label and location, between an unlearned pattern and its classification neighbourhood as the judgment basis of whether a pattern is selected as a prototype. In addition, PSNB adds a collective dynamic updating process of *TP*, and utilizes the change of classification neighbourhood distances and the neighbourhood retrospect to identify the change of classification boundary as the judging basis to judge the boundary change of the prototype, so as to retain the classification boundary decision prototype and remove the prototype far away from boundary zone and even outlier, thus achieving the collective dynamic updating of *TP*.

Its main treatment strategy is as follows: First, aims at the deficiency of the simple prototype absorption processing strategy of traditional CNN, which is vulnerable to the interference of outlier, noise and other abnormal patterns, and resets the prototype absorption strategy when the pattern to be learned is of the same category as the nearest prototype, retaining each pattern with high boundary approximation as new prototype and reducing the noise sensitivity. Second, referencing the group judgment and the noise process, PSNB makes full use of the neighbourhood boundary distance of each prototype and the class label information of the tracking prototypes to recognize the location information of the relevant prototypes in the learning process, and then makes necessary identification. Finally, appends the updating process. PSNB introduces an adaptive updating threshold according to the classification boundary spacing and update identification information. When meet the updating condition, according to update the adaptive threshold to delete the lower boundary contribution of prototype, retain high boundary contribution of prototype, adaptive step by step implementation classification boundary approximation, and ensure the prototype dynamic selection of classification boundaries.

### Algorithm process

Taking into account the definitions given in Section 3, utilizing the useful information obtained by the neighbourhood of an unlearned pattern, pattern location, noise even outlier can be recognized. From the neighbourhood relationship, PSNB manages to whether an unlearned pattern is selected as a prototype and judges whether the prototype is redundancy. Then PSNB can select the prototypes whose classification distance is better than its nearest prototypes and eliminate those interfere prototypes in order to maintain the high decision *TP*.

The application of *PS* integrated editing and condensing has some additional properties with regard to the conventional algorithms: First, they consider the shape of neighbours as a variable feature which depends on the requirements of the specific application. Second, since the classified neighbourhood of an unlearned sample or a prototype always tends to surround classification boundaries, the information extracted from prototypes close to classification boundaries, where uncertainty is highest, may be richer in the sense of the distribution of prototypes.

After computing the nearest neighbourhood of each prototype in *TP*, all the neighbour prototypes of an unlearned pattern are obtained. Through traversing the nearest neighbour relationship down from each prototype, its location, its boundary distance, etc, are calculated. Using the information, these prototypes of high classification contribution are reserved and those prototypes of low classification contribution are removed based on the similar strategy nearest neighbour editing strategy.

In other words, all prototypes that are surrounding each prototype take part in the process of estimating whether it is an outlier or not, regardless of their actual distance and location to the prototype. The approach to this ensemble *PS* scheme can be expressed in the following way.
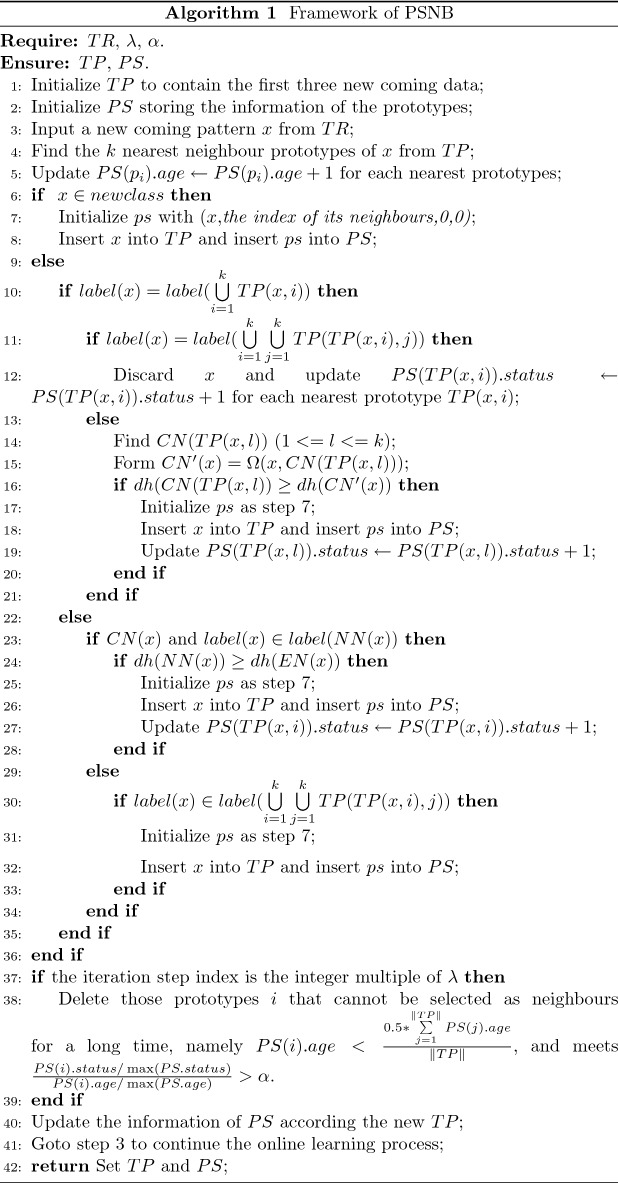


In Algorithm 1, *TP* is initialized with the first three patterns from *TR* and the nearest neighbour information of *PS* based the initialize *TP* is set synchronously. The updating status and age of each node in *PS* is 0 initially. So, when a pattern is selected as a prototype in any situation, *TP* and *PS* are adjusted at the same time. Therefore, a pattern is inserted into *TP*, the new generating quintuple ps of the new prototype is assigned with the corresponding values and inserted into *PS*.

For an unlearned pattern *x* that comes from data stream or static dataset, if *x* comes from a new class, *x* is took as a prototype and the *k* nearest neighbours from *TP* are found to set these corresponding values of the new generated structure *ps* of *x*. The remaining values of *ps* are setting as 0. *x* and its information structure *ps* are inserted into *TP* and *PS*, correspondingly. Otherwise, the *k* nearest neighbours of *x* are found and the ages of those nearest neighbours are updated by adding 1. When *NN*(*x*) is homogeneous (namely these nearest neighbours have the same class label) but the class label of *x* is not same with *NN*(*x*), *x* is selected as a new prototype and the above same operation of *TP* and *PS* are performed. If some prototype of *x*’s nearest neighbours has the same class with *x*, some new classify boundary subset of *x* is consisted by replacing some nearest neighbour prototype which has the same class by *x* in *NN*(*x*). If exist the classify distance of a new consisted subset is smaller than the distance of *CN*(*x*), *x* can be selected as a new prototype.

Otherwise due to *NN*(*x*) is heterogeneous and might have the different class label with *x*, *x* maybe a noise. So, further judgment is needed to do. If some offspring nearest neighbours have the same class label with *x*, *x* is selected as a new prototype and the same above operation for *TP* and *PS* is performed; otherwise, *x* is a noise and discarded.

If the above insertion conditions donot satisfy, *x* is not prototype, even some prototypes are redundancy. To redundant prototypes, it is necessary to find them and mark them by increasing their updating values. Whether *x* is a prototype or not, its nearest prototypes are active and most relevant with *x*. The active and relevant information should be maintained. In the paper, the age value is used to represent the selected times of a prototype that is took as one of *k* nearest neighbours of new coming patterns. When a prototype is selected as one of *k* nearest neighbours of a new pattern, the age value of the prototype pluses 1.

## Algorithm evaluation

The properties and performance of PSNB are obseved form two types of experiments, i.e., the boundary approximating experiments and the benchmark dataset experiment.

### Artificial data experiments

In order to verify the effective reduction performance of PSNB and its retention ability to the original distribution, this paper selects three artificial data sets with different distribution to demonstrate the reduction results of PSNB.

#### Boundary approximating experiments

**Figure 3:**
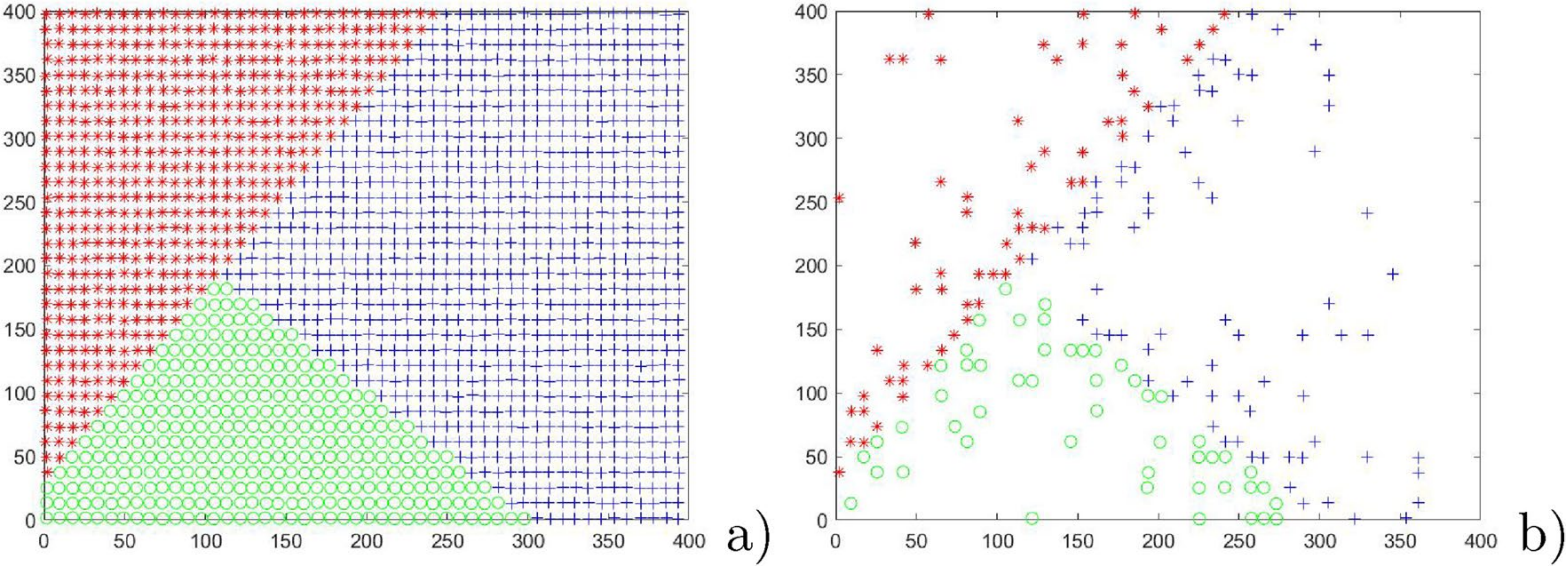
The reduction result of linear boundary dataset.

**Figure 4:**
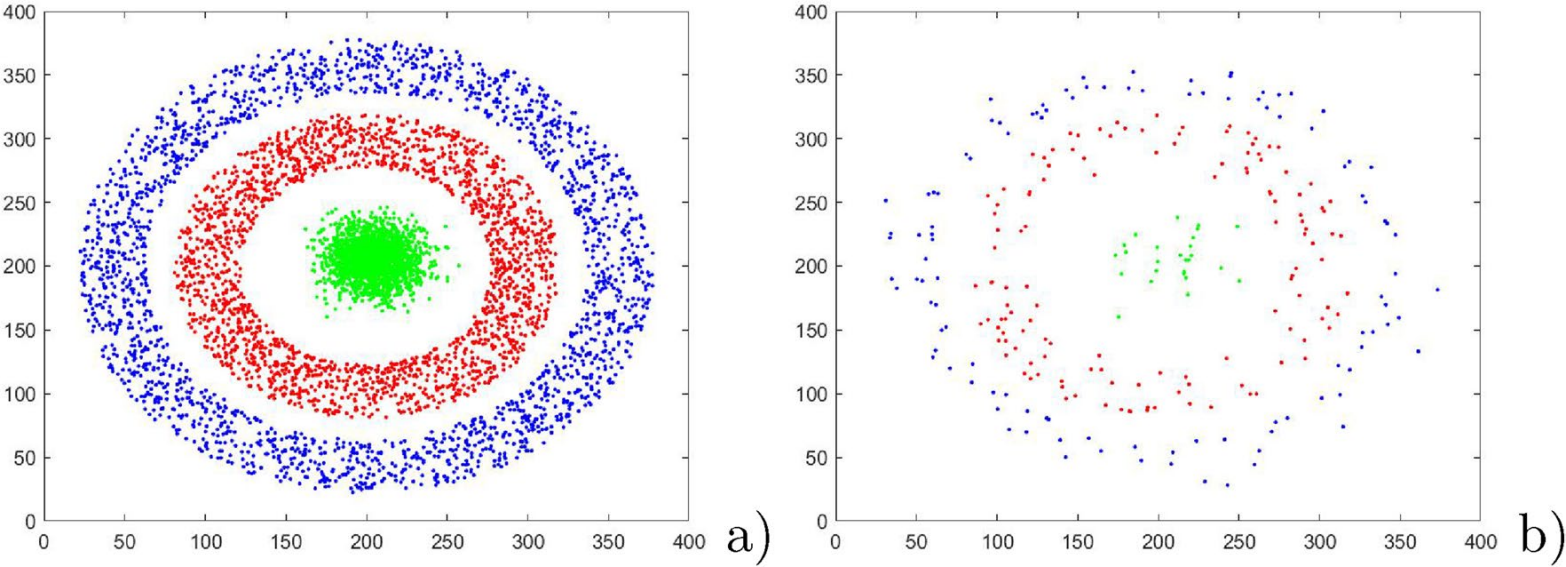
The reduction result of circular boundary dataset.

**Figure 5:**
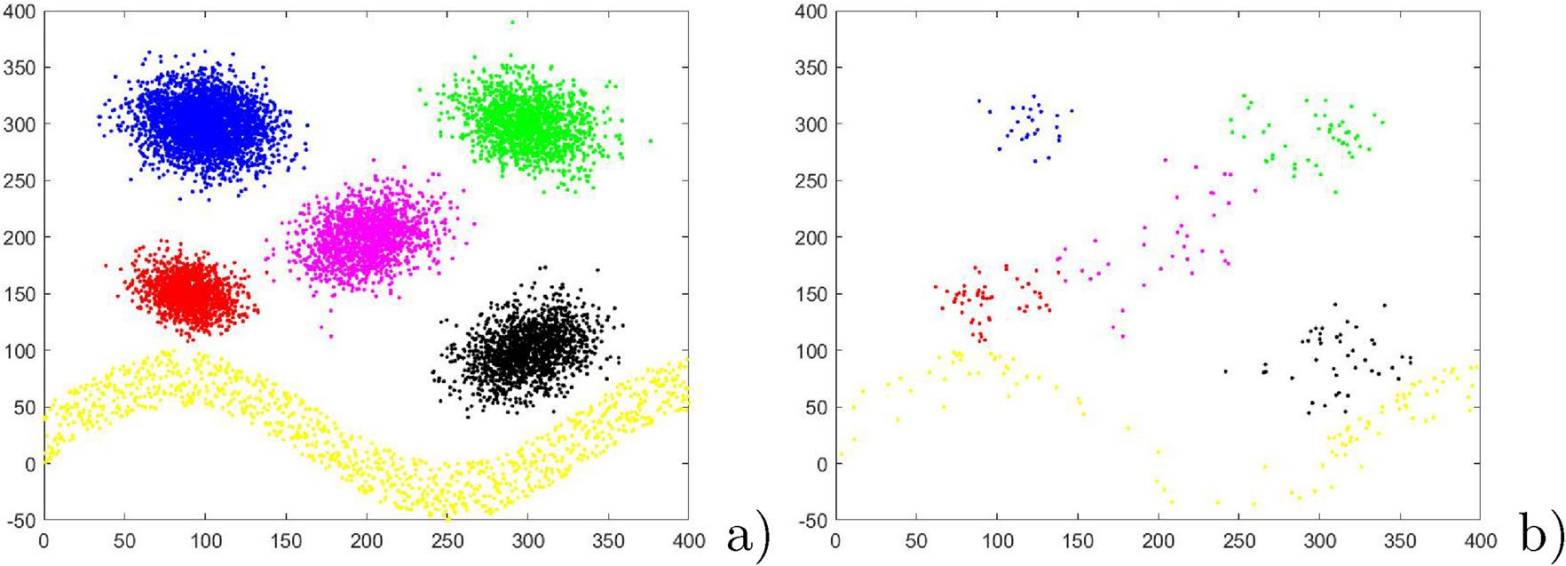
The reduction result of dataset with gaussian and sinusoidal distribution.

In this section, the experiment is conducted in three two-dimensional datasets, as showed in Figs. 3a, 4a and 5a. First, Figs. 3a, 4a and 5a are applied to show the performance of PSNB to handle different distribution: Fig. 3a 1700 patterns agree with two-dimension uniform distribution with perturbation within [0,1] and have clear linear boundaries; Fig. 4a 6000 patterns are evenly divide to three classes in which Class 1 and Class2 are two concentric circles, and Class 3 are a Gaussian distribution; Fig. 5a 10000 patterns are divided to six class labels, in which all of Class 1–5 meet the same distribution as two-dimensional Gaussian distribution and Class 6 is a sine distribution. For PSNB, the parameters is set as $\alpha =2$ and input patterns are obtained by a random sequence from artificial dataset. Then Fig. 3b, 4b and 5b indicate the reduction results of PSNB.

From Fig. 3, each class is represented very well by adaptively selected and updated prototypes: the boundary is maintained effectively even though the single pass random sequence has produced some negative effects. To within class region, PSNB can effectively reduce the number of prototypes. To between class region, PSNB is basically preserved the real classification boundary. From Fig. 4, the outermost ring is represented very concisely. The inner ring is represented graphically by a few of prototypes that clearly represent the ring boundary. The innermost Gaussian distribution patterns are noticeably sparse that retains the Gaussian distribution original shape. From Fig. 5, except for the similar reduction result for these Gaussian distribution patterns as that of Fig. 4b, the sine boundary is maintained while the patterns being reduced.

#### Threshold sensitivity experiments

In the updating process, there are two parts to influence the reduction ratio. The first part is to delete these prototypes without selected as neighbours. The second part is to delete some redundancy. The threshold $\alpha$ is adopted to remove those redundant prototypes and control the scale of *TP*.

In general, when a prototype has high age time and low updating value at the same time, it is deemed as an activity decision prototype and should be retained. In other word, it is common that a prototype has high age time and low updating value. Rather, it should be eliminated. The contradictions that a prototype has high age times and high updating times rarely occur due to the period updating process. Then the contradiction is neglected in this paper. Besides, there are many prototypes that fell between the above cases. To these prototypes, the threshold value $\alpha$ indicates the focus of the removement. Different value of $\alpha$, different reduction ratio and different classification accuracy. The smaller $\alpha$ the greater the force of removement. The closer the value of $\alpha$ is to 0, the updating status plays the greater role in the reduction process. It is not allowed that the majority, even all the prototypes will be deleted. On the contrary, the bigger value of $\alpha$ indicates the poor role in the updating process. Meanwhile, considering the relationship between the age time and the updating status, the value of $\alpha$ be larger than 1 with less than 10 is appropriate. For further comparison, the value of $\alpha$ is still set to 0.5.

**Figure 6:**
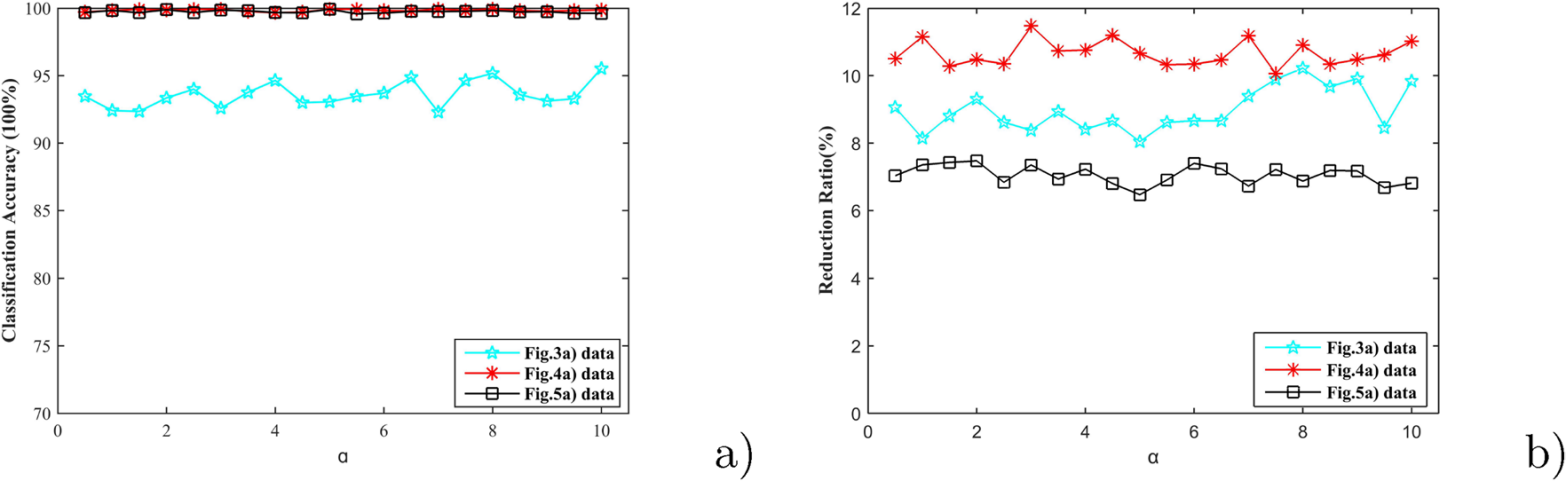
The results with different updating threshold value.

To give a detail comparison, KNN is selected as the reference algorithm. The value *k* is set by 1, 3, 5, 7, 9. The average classification accuracy percentages of Fig. 3a, 4a and 5a are 99.81, 99.98, 99.96, 99.85 and 89.61 respectively. PSNB obtains the classification accuracy and reduction ratio under 5-fold cross-validation and random sequence based on the same datasets with the reference algorithm KNN, that is shown in Fig. 3a, 4a and 5a. The results of both classification accuracy and reduction ratio are little change. It is clear that the classification accuracies are not worse than that of KNN. However, relatively speaking, the change of reduction ratio is more obvious than that of classification accuracy. Even to the most volatile reduction ratio in Fig. 6b, its variance is 0.67 while its mean is 9.0544. It can still be thought that the reduction ratio is insensitive to $\alpha$. Although some fluctuations from Fig. 6, the fluctuations are caused by 5-fold cross-validation and random sequence. On the whole, the whole reduction ratio has little effect to the updating threshold $\alpha$. In other words, PSNB is insensitive to the updating threshold. The results from Fig. 6 show this conclusion as well.

### UCI benchmark dataset experiments

To evaluate the proposed algorithm better, it is compared to some famous prototyped-based algorithms. Meanwhile, KNN is selected as the benchmark of the classification accuracy of these compared algorithm. The average results of KNN are obtained under $k=1,3,5,7,9$. In addition, several datasets from the UCI repository of machine learning databases^36^ (http://archive.ics.uci.edu/ml/) are selected to verify the classification performance of the compared algorithms. Three kinds of results are obtained: classification accuracy (*Acc*), reduction ratio (*Str*) and running time. To the prototype-based algorithms, the classification accuracy is the result using 1-NN and *TP* as decision dataset. The reduction ratio is the percentage of *TP* in *TR* and computed as $Str=100*N/n$^28^. Due to the insensitive of the proposed algorithm, $\alpha$ of PSNB is set to 2. All the average results are obtained under five-time 5-fold cross-validation policy.

#### Imbalance dataset experiments

In this section, seven datasets of Glass, Ecoli, Balance, Yeast, Ctg, Tuandromd and Page in UCI are used to test the ability of the proposed algorithm to cope with imbalance dataset. The overall condition of these data sets is shown in Table 1. The overall results of these datasets are shown in Table 2.

**Table 1:** Overview of the imbalance datasets in the experiments.

Dataset	Number of patterns	Number of classes	Number of features	Ratio of original class distribution
Glass	214	6	9	(32.71:35.51:7.94:6.07:4.21:13.55)
Ecoli	336	8	7	(42.56:22.92:0.60:0.60:10.42:5.95:1.49:15.48)
Balance	625	3	4	(7.84: 46.08:46.08)
Yeast	1484	10	8	(16.44:28.91:31.20:2.96:3.44:10.98:2.36:2.02:1.35:0.34)
Ctg	2126	3	20	(7.76:52.45:39.79)
Tuandromd	4464	2	241	(79.86:20.14)
Page	5473	5	10	(89.77:6.01:0.51:1.61:2.10)

From Table 2, for these imbalance datasets, despite the extremely low reduction ratio to each imbalance dataset, PSNB can maintain all sort of class prototypes, even rebalance the class distribution to strengthen the refulgence of minor classes. The percentages of the minority classes are improved in addition. In other words, the proposed algorithm weakens the unbalance. Although the extremely low reduction ratios of each data sets, the classification accuracies are in close proximity to that of KNN. So PSNB has good reduction efficiency and the ability to process imbalanced data set.

**Table 2:** The results of the imbalance datasets in the experiments.

Dataset	KNN(*Acc*)	PSNB(*Acc*)	PSNB(*Str*)	Ratio of final class distribution
Glass	66.63	62.46	14.43	(40.93:31.36:8.22:6.39:4.52:8.58)
Ecoli	84.22	78.46	13.85	(29.28:27.92:0.93:0.85:15.70:6.21:1.69:17.41)
Balance	83.61	79.62	9.01	(8.11:55.03:36.86)
Yeast	54.85	48.89	5.56	(13.83:28.93:34.62:3.52:2.58:8.15:3.19:1.98:1.93:1.27)
Ctg	82.49	71.78	4.54	(7.95:51.29:40.76)
Tuandromd	96.88	98.69	9.67	(47.69:52.31)
Page	95.56	91.93	3.18	(83.55:8.73:1.00:2.80:3.92)

#### Stationary experiments

Here, the famous prototype-based algorithms of CNN, ENN, PSC, and TRKNN are selected as compared algorithms. CNN and ENN are the basis of the current improved algorithms. PSC and TRKNN are efficient boundary recognition algorithms. All the above algorithms except CNN are unable to cope with incremental learning tasks. The UCI datasets of Iris, Liver, HillValley, Pima, Segment, Sat, and Pendigits with different scales to test the proposed algorithm. All the whole scales of datasets are not large. For the above reason, the experiment is running in stationary environment in this section. Random sequence is also adopted to tune the parameters in each algorithm. The overall condition of these data sets is shown in Table 3. KNN still is a benchmark algorithm as above. According to the literatures^28,31^, PSC takes the best parameters $c=6r$ and $c=8r$, TRKNN using $\alpha =1.2$ and $\alpha =1.4$. In addition, ENN takes 3, 5, 7, 9 as *k* value. All the results shown in Table 4 are the average results obtained by compared algorithms.

**Table 3:** Overview of the datasets in stationary experiments.

Dataset	Number of patterns	Number of classes	Number of features
Iris	150	3	4
Liver	345	2	6
HillValley	606	2	100
Pima	768	2	8
Segment	2310	7	19
Sat	4435	6	36
Pendigits	9849	10	16

From Table 4, for Iris, Pima, and Segment, the classification performance of PSNB is best or nearly the results of KNN that are deemed as the best results. For all databases, the average classification accuracy of PSNB is higher than those of all other algorithms excluding KNN. PSNB has the best classification accuracy than all the others on Hillvalley. For Pendigits, PSNB works slightly worse than CNN but better than other algorithms. For Liver and Sat, although the higher classification accuracies of PSNB are better than that of other algorithms, the gaps between them and the best are a little big. To the aspect of classification accuracy, PSNB is superior to other compared algorithms.

**Table 4:** Classification accuracy of stationary experiments.

Dataset	KNN	CNN	ENN	PSC	TRKNN	PSNB
Iris	96.38	94.67	90.40	93.86	86.52	96.01
Liver	64.39	52.05	59.88	56.07	56.78	60.58
HillValley	54.53	56.08	54.34	49.67	51.13	68.09
Pima	71.25	61.31	68.15	54.85	64.86	67.19
Segment	94.02	93.67	77.32	80.94	76.63	94.59
Sat	90.24	79.48	63.99	70.48	62.55	84.42
Pendigits	99.13	98.63	84.95	83.02	88.49	98.31
Average	81.42	76.56	71.29	59.84	69.56	81.31

Table 5 shows the average reduction ratio of PSNB is remarkably better than that of other algorithms. For Iris, the reduction ratios of PSC and PSNB are less than 20%, which are clearly lower than other algorithms. For Liver, TRKNN is slightly better than PSNB, meanwhile PSNB is better than CNN, ENN and PSC. For Pima, Segment, and Sat, PSNB has clear advantages over other algorithms. For Pendigits, although PSNB is worse than ENN, it can obtain the lower ratio than others. However, PSNB has the worst reduction ratio on Hillvalley.

**Table 5:** Reduction ratio of stationary experiments.

Dataset	KNN	CNN	ENN	PSC	TRKNN	PSNB
Iris	100	58.54	51.12	23.89	30.56	22.67
Liver	100	21.37	53.69	43.48	10.86	12.93
HillValley	100	50.82	55.63	23.18	13.24	59.42
Pima	100	17.41	61.29	21.13	37.22	10.65
Segment	100	67.75	16.55	43.12	22.91	13.91
Sat	100	25.79	43.35	15.17	25.81	12.73
Pendigits	100	38.43	12.04	26.35	30.18	15.11
Average	100	40.06	41.95	26.19	24.39	21.07

Moreover, comprehensive comparison of Tables 4 and 5, for Iris, Segment, and Sat, PSNB performs well in both classification accuracy and reduction ratio. For Pima, although PSNB cannot obtain the higher classification accuracy than ENN and the lower reduction ratio than TRKNN, PSNB has the best average performance than other algorithms. For Liver and Pendigits, the results further verify that PSNB can efficiently obtain the higher classification accuracy under the lower reduction ratio. For Hillvalley, PSNB can obtain the highest classification accuracy under the highest reduction ratio. Although PSNB has the highest reduction ratio on Hillvalley, it shows that PSNB has the ability to handle high-dimensional dataset.

**Figure 7:**
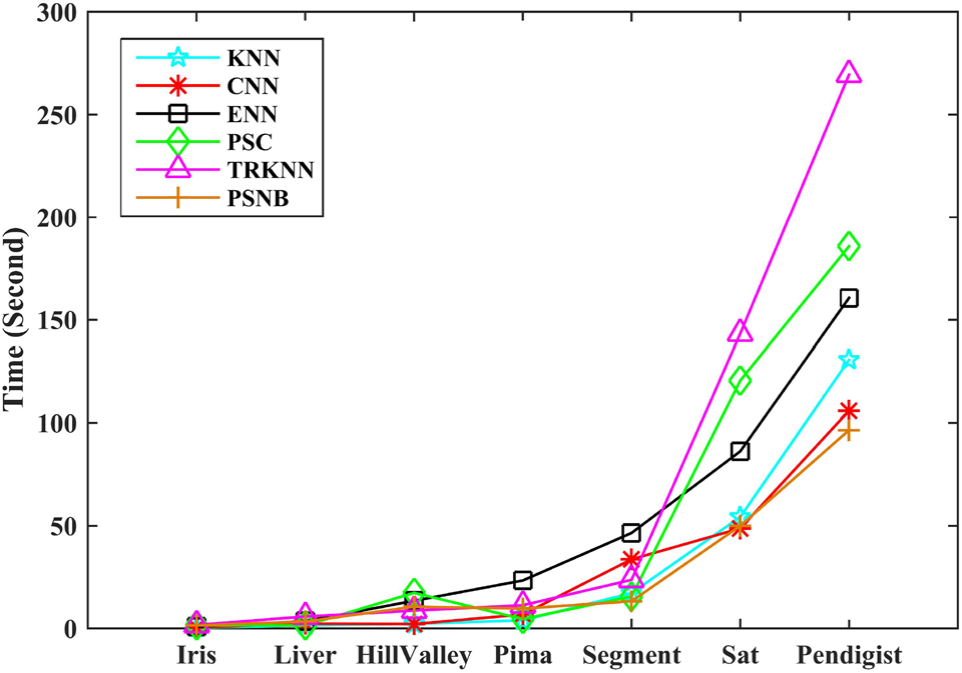
Running time of stationary experiments.

Figure 7 shows the average running time of the compared algorithms. For KNN, the running time is the entire classification time. For other algorithms, the running time includes two part: the time to obtain the decision prototype set and the classify time using the decision prototype set. The time graph clearly shows that all compared algorithms have the little bit closer running times in small size dataset, such as Iris, Liver, Hillvalley and Pima. For Segment, TRKNN and PSNB has significantly longer running time due to their dividing or heterogeneous-chain building mechanisms. For Sat and Pendigits, PSNB embodies its fast advantage.

#### Incremental experiment

Here, another experiment is conducted in an incremental environment in order to illustrate another advantage of PSNB. Similar to Section 4.1, the incremental environment is simulated by single pass random sequence. So, the results of Section 4.1 are the results of incremental experiments. In order to further test the efficiency of PSNB, a special test is applyed to compare with an incremental algorithm. The UCI dataset, Letter, is selected to test the proposed algorithm to deal with incremental environment. Letter contains 20000 patterns with 26 classes and 16 features. As an incremental prototype generation algorithm, ILVQ is famous for its low reduction ratio and its high classification accuracy. Meanwhile, ILVQ can maintain the classification boundary in spite of some expansion of classification boundary is existed seldomly. So ILVQ is selected as compared algorithm and set $\lambda =AgeOld=\sqrt{n}$^6^. Take the same running policy, ILVQ and PSNB achieve the average results that are listed in Table 6.

**Table 6:** The results of incremental experiment.

Algorithm	*Acc*	*Str*	*Time*
KNN	95.41	100	202.37
ILVQ	86.02	18.23	124.69
PSNB	91.16	15.64	138.73

From Table 6, it is apparent that the performance of PSNB in Letter is at least no less than the compared algorithms. PSNB can obtain higher classification accuracy than ILVQ. It means PSNB has a clear advantage than ILVQ. The same conclusion is also true to reduction ratio because PSNB has lower reduction ratio than that of ILVQ. The lower reduction ratio means less storage space requirement. The lower reduction ratio refers to the small scale of decision prototype set *TP* and the time to label unknown patterns. So PSNB can adapt to incremental learning tasks. Because PSNB needs to rebuild the new neighbour relationship each updating iteration, it needs more running time than ILVQ. Even so, PSNB is still deemed as a fast prototype selection algorithm like ILVQ.

## Conclusion

As described in this paper, the proposed incremental prototype selection algorithm PSNB grows gradually and stores the selected prototypes perfectly so that it can realize the incremental learning task well. Moreover, a proper updating scheme is taken to ensure PSNB to eliminate the noise and sparse the prototypes that locate at the central class region during selection. It takes advantage of an updating threshold criterion to automatically control the number and the quality of prototype. PSNB is insensitive to threshold and adaptive to datasets of different distribution. Consequently, predetermining different appropriate threshold values have little effect on the reduction ratio and classification accuracy. In the experiments, PSNB is compared with some famous prototype-based algorithms. The results show that PSNB can obtain high decision set of small scale and well adapt to complex data distribution including noise. It further verifies that PSNB is insensitive to the updating threshold with suitable range although preset and specified threshold value can determine the update intensity. In addition, the reduction ratio of PSNB is better under most conditions. In respect of recognition ability, the performance of PSNB is also good. Although some algorithms such as ENN achieve a better classification accuracy, it must be emphasized that PSNB fulfills the best overall efficiency between classification accuracy and reduction ratio. Moreover, experiments demonstrate that PSNB can perform in the incremental environment well.

## Data Availability

The source code and the datasets generated and/or analysed during the current study are available from the corresponding author on reasonable request.
